# Teaching lesbian, gay, bisexual and transgender health in a South African health sciences faculty: addressing the gap

**DOI:** 10.1186/1472-6920-13-174

**Published:** 2013-12-27

**Authors:** Alexandra Müller

**Affiliations:** 1 School of Public Health and Family Medicine, Health Sciences Faculty, University of Cape, Town, Anzio Rd, Observatory, Cape Town 7925, South Africa

**Keywords:** LGBT health, Health professions education, Heteronormativity, Homophobia, South Africa

## Abstract

**Background:**

People who identity as lesbian, gay, bisexual and transgender (LGBT) have specific health needs. Sexual orientation and gender identity are social determinants of health, as homophobia and heteronormativity persist as prejudices in society. LGBT patients often experience discrimination and prejudice in health care settings. While recent South African policies recognise the need for providing LGBT specific health care, no curricula for teaching about LGBT health related issues exist in South African health sciences faculties. This study aimed to determine the extent to which LGBT health related content is taught in the University of Cape Town’s medical curriculum.

**Methods:**

A curriculum mapping exercise was conducted through an online survey of all academic staff at the UCT health sciences faculty, determining LGBT health related content, pedagogical methodology and assessment.

**Results:**

127 academics, across 31 divisions and research units in the Faculty of Health Sciences, responded to the survey, of which 93 completed the questionnaire. Ten taught some content related to LGBT health in the MBChB curriculum. No LGBT health related content was taught in the allied health sciences curricula. The MBChB curriculum provided no opportunity for students to challenge their own attitudes towards LGBT patients, and key LGBT health topics such as safer sex, mental health, substance abuse and adolescent health were not addressed.

**Conclusion:**

At present, UCTs health sciences curricula do not adequately address LGBT specific health issues. Where LGBT health related content is taught in the MBChB curriculum, it is largely discretionary, unsystematic and not incorporated into the overarching structure. Coordinated initiatives to integrate LGBT health related content into all health sciences curricula should be supported, and follow an approach that challenges students to develop professional attitudes and behaviour concerning care for patients from LGBT backgrounds, as well as providing them with specific LGBT health knowledge. Educating health professions students on the health needs of LGBT people is essential to improving this population’s health by providing competent and non-judgmental care.

## Background

Identifying as lesbian, gay, bisexual or transgender (LGBT^a^, for a complete terminology see Table 1) is not genetically or biologically hazardous, but societal homophobia confers risk factors for LGBT people’s well being. Homophobia, the irrational fear and hatred of people who are attracted to the same sex, leads to social exclusion, experiences of discrimination and stigma, and in the worst case to violence directed against people whose real or perceived sexual orientation does not fit the narrowly defined heterosexual norms [1]. For example, in Southern Africa sexual violence against lesbian women, often motivated by homophobia, places them at an increased risk for HIV infection [2]. Gender identity and sexual orientation, like other social determinants of health, lead to health disparities and, compared with heterosexual and non-transgender socioeconomically matched peers, LGBT individuals are more likely to face barriers accessing appropriate health care [3]. Beyond these societal risk factors, LGBT people have specific health and health care needs in various fields from chronic disease risk [4], adult and adolescent mental health [5], unhealthy relationships (for example intimate partner violence [6]), sexually transmitted infections [7], and human immunodeficiency virus infection [8]. Data on sexual orientation and gender identity is often not collected in large population surveys; however, recent data from the United States gives a good indication of the prevalence of LGBT identities: 3.8% of Americans identify as lesbian, gay, bisexual or transgender, and even more (8.2%) have had same-sex sexual experiences or experienced same-sex attraction (11%) [9]. It is therefore very likely that every health professional will encounter patients with LGBT health needs in the course of his or her career.

**Table 1:** List of terms and abbreviations related to LGBT health*

	
**Bisexual**	Refers to people who are emotionally, physically and sexually attracted to people of both sexes.
**Gay**	Refers to men who are emotionally, physically and sexually attracted to men.
**Gender**	Is a socially constructed system of classification that ascribes qualities of masculinity and femininity to people. Gender characteristics can change over time and are different between cultures.
**Gender identity**	Is one’s basic sense of being male or female or another gender. It usually, but not always, matches the sex based on the external genitalia present at birth.
**Heterosexism**	Is the assumption or belief that everyone is and should be heterosexual and that other sexual orientations are unhealthy, unnatural and a threat.
**Heterosexual**	Refers to people who are emotionally, physically and sexually attracted to people of the opposite sex.
**Homophobia**	Is the irrational fear of, hatred against, or disgust towards homosexuals or homosexuality.
**Homosexual**	Refers to people who are emotionally, physically and sexually attracted to people of the same sex.
**Lesbian**	Refers to women who are emotionally, physically and sexually attracted to women.
**MSM**	Is an abbreviation for men who have sex with men, a term often used in public health. MSM do not necessarily identify as gay or bisexual.
**Sex**	Is commonly understood as the classification of a person as male or female at birth, based on bodily characteristics such as chromosomes, hormones, internal reproductive organs, and genitalia.
**Sexual orientation**	Refers to whom people are intimately attracted to. People can be attracted to members of the same sex, of the opposite sex, or both. Western society tends to think of sexual orientation as expressing itself in three forms: homosexual (gay or lesbian), heterosexual (sometimes referred to as ‘straight’) or bisexual (having both homosexual and heterosexual feelings). People also identify as queer (refusing to fit into any category) and asexual (not being sexually attracted to people).
**Transgender**	Refers to people whose gender identity is different from the sex assigned at birth. A transgender person may choose to adhere to the gender role with which that person identifies. A person who does not adhere to gender roles is called gender non-conforming. It is important to recognise that the gender binary (the view that people are either male or female) does not describe the identity of many people.
**WSW**	Is an abbreviation for women who have sex with women, a term often used in public health. WSW do not necessarily identify as lesbian or bisexual.
	**Excerpts from: *Understanding the Challenges facing Gay and Lesbian South Africans*, available from OUT LGBT Pretoria and *Guidelines for primary care workers providing care for transgender patients*, available from Gender Dynamix, Cape Town**

*For more information visit http://www.out.org.za, http://www.triangle.org.za, or http://www.genderdynamix.org.za.

Health professionals are not immune to prejudice and many LGBT patients experience health care as an unsafe space, mostly because of the attitudes of doctors and nurses [10,11]. LGBT patients perceive health professionals’ disdain, which alienates them from the medical system and reduces their utilisation of screening modalities, risking higher morbidity and mortality from infections, cancers, and heart disease [12]. Negative experiences with health professionals contribute to the erosion of a sense of safety in the health care system, and as a consequence, LGBT people avoid seeking care [13]. A 2006 South African study highlights the alarming consequences for LGBT people’s health-seeking behaviour: in the province of the Western Cape, 16% of LGBT people either delayed seeking health care for fear of homophobic treatment, or did not seek medical help at all [14]. The fear of homophobic treatment is often justified: recent reports from various South African contexts document that gay men, lesbian women and transgender people are discriminated against, insulted, and sometimes even refused health care when accessing HIV services [15-18].

Heteronormativity invisibilises LGBT people, whose lived realities are not reflected in mainstream narratives, and as a consequence, LGBT patients either hide part of their identity from their health care provider, or have to educate health professionals about their sexual orientation or gender identity [1]. In South Africa, recent years have brought a shift to include LGBT health into health care policy recommendations. For example, transgender people are identified as one of the most-at-risk populations in the 2012–2016 National Strategic Plan for HIV, STIs and TB [19]. Furthermore, the first South African National Health Assembly, held in Cape Town in June 2012, called for ‘appropriate non-judgmental care for marginalised vulnerable groups such as […] LGBT persons’ [20]. In the United States, the report on LGBT health by the Institute of Medicine points out that a more solid evidence base about LGBT people’s health is the first step to addressing the challenges around LGBT people’s health needs [21]. An important first step towards equipping future health professionals to provide competent care to LGBT patients is to ensure that teaching LGBT health related content is systematically integrated into the health sciences curricula [22]. The Association of American Medical Colleges (AAMC) has recommended that ‘medical school curricula ensure that students master the knowledge, skills, and attitudes necessary to provide excellent, comprehensive care for [LGBT] patients’ by including ‘comprehensive content addressing the specific healthcare needs of [LGBT] patients’ and ‘training in communication skills with patients and colleagues regarding issues of sexual orientation and gender identity’ [23]. Teaching LGBT health should challenge heteronormativity and pervasive stereotypes about gender and sexuality, sensitise students to the harmful effects of stigmatisation, and provide information about the specific health issues and challenges that LGBT people face [1].

In the South African health sciences, the dominant pedagogical approach to sexuality is biomedical and developmental, with almost no space for an interrogation of the constructions of gender and sexuality through social dynamics [24]. Sexuality rarely features in analyses of power relationships, health access, health systems management, or epidemiology, but is mostly taught as ‘reproduction’, sometimes related to ‘risk’ or ‘dysfunction’, and not as a predictor of health ^[ibid]^. Contrary to a primary health care approach, the biomedical and pathological aspects of sexuality are isolated from its social context, people’s sexuality is reduced to anatomical (ab)normalities and physiological (dys)functioning, and their sexual identities, desires, pleasures and orientations are ignored ^[ibid]^. Bennett and Reddy^[24: 249]^ conclude that ‘health science courses hold enormous potential for thinking through a critical analysis of gendered relations and a sophisticated understanding of sexualities’, and that ‘the intellectual and practical expertise introduced through health sciences hold potential for excellent gender and sexualities education, despite the current positivist and biomedical orientation of the curriculum’.

At the University of Cape Town (UCT), the current MBChB curriculum does not provide a systematic approach to LGBT health related topics. In 2012, the health sciences faculty’s MBChB Curriculum Revision Task Team (CRTT) agreed to investigate possibilities for teaching LGBT health related content. To inform this process and ultimately create a curriculum for teaching gender and sexuality across the health sciences, beginning with the MBChB programme, I mapped how LGBT health related content is currently taught across the health sciences faculty. The aim of the curriculum mapping was to evaluate current teaching content and identify gaps and repetition on LGBT health related topics. It also aimed to identify existing teaching resources and lecturers who possess special knowledge on the topic. This was a first step towards building synergies and possible teaching collaborations, and creating a comprehensive curriculum for LGBT health related content that could provide a model for health sciences faculties across South and Southern Africa. I am sharing the outcome of this curriculum review, as I believe that it highlights a crucial gap, and opportunity, in the education of medical and other health professions students.

## Methods

To ascertain when and how academic staff teach LGBT health in UCT’s health sciences faculty, I mapped the current curricula through an online survey. To reach all lecturers and educators who teach in the health sciences faculty, invitations were sent through UCT’s email system and the health sciences faculty listserv. In addition, staff members who had been identified as currently teaching issues of gender and sexuality, or were located in key departments, were directly contacted. Staff were asked to participate in an internet-based, self-administered survey. This online survey allowed me to gather a meaningful amount of data over a short time, and thus provide a cross-sectional view of all staff that participated in the survey. It consisted of 10 questions and asked if, where and how the respondents teach topics of gender and sexuality in the curriculum of MBChB, Occupational Therapy, Physiotherapy, Communication Sciences & Disorders as well as Nursing and Midwifery. The survey asked about topics relating to the theory of sexuality and gender, specific diseases and health conditions, as well as about the impact of gender and sexuality on health-seeking behaviour and access to care (adapted from Obedin-Maliver 2011 [22], see Table 2 for a list of LGBT health topics). In addition, it asked about how learning outcomes were assessed, and whether the respondents recommended any other topics relating to gender and sexuality that should be taught. The survey ran for 14 days, and reminders were sent out via email on day 8 and 11. All answers were collected with the online survey tool Survey Monkey and analysed further using Microsoft Excel 2011. Staff who had completed the survey had the option to provide an email address to receive the survey’s results in the form of a report. The study was approved by the Faculty’s Human Research Ethics Committee (reference 537/2012), and respondents agreed to participate by reading and accepting an online informed consent form.

**Table 2:** LGBT health topics, number and division of respondents teaching

***Topic***	***n***	***Division***
Definition and theories of sexual orientation	4	HUB*, Psychiatry, paediatrics, Medicine
Homophobia, heterosexism	1	Medicine
Barriers to access to health care for LGBT people	4	PHC, Medicine, psychiatry, family medicine
Alcohol, tobacco, or other drug use for LGBT people	0	-
Safer sex for LGBT people	0	-
HIV in LGBT people	2	CLS †, Medicine
Sexually transmitted infections (not HIV) in LGBT people	2	CLS, Medicine
Chronic disease risk for LGBT populations	0	-
Disorders of sex development (DSD)/Intersex	3	HUB, Paediatrics
Transitioning (e.g. male-to-female, female-to-male)	6	HUB, PHC ‡, Family medicine, paediatrics, psychiatry
Sex reassignment surgery (SRS)/Gender affirming treatment	4	HUB, Paediatrics, Psychiatry
LGBT adolescent health	0	-
Mental health in LGBT people	0	-

*HUB: Human Biology; †CLS: Clinical Laboratory Sciences; ‡ PHC: Primary Health Care Directorate.

## Results

A total of 127 UCT academic staff, from 31 different divisions and research units, responded to the call for participation and visited the survey information site. Of these, 116 consented to participate in the survey. Of the 116 respondents who consented, 93 proceeded to the survey and provided information. A wide range of teaching units responded to the survey, including preclinical (e.g. Human Biology, Public Health), clinical (e.g. Paediatrics, Surgery) and allied health sciences (Table 3). Only 10 respondents taught LGBT health-related topics as outlined in Table 2. Forty-one respondents requested a report of the outcomes after completing the survey.

**Table 3:** Respondents to the survey

***Curriculum***	***Total respondents***	***Respondents teaching LGBT health***
MBChB preclinical	29	5
MBChB clinical	32	5
Nursing & midwifery	5	-
Occupational therapy	12	-
Communication sciences and disorders	4	-
Physiotherapy	7	-
Audiology	4	-

### Teaching content

There was no course dedicated exclusively to teaching LGBT health. However, certain issues of LGBT health content were taught within various existing courses within the MBChB curriculum, ranging from Human Biology to Psychiatry (Table 2). No LGBT health related teaching was conducted within the allied health sciences. Materials related to LGBT safer sex, mental health, substance use, chronic disease risks, and adolescent health were not taught at all. One lecture on HIV in men who have sex with men also addressed heteronormativity and homophobia. Health system concerns about LGBT health, notably access to health care (n = 4), as well as disorders of sex development (n = 3), transitioning (n = 6) and gender reassignment surgery (n = 4) were well covered. No course or lecture addressed medical students’ own knowledge, attitudes, prejudices and beliefs about sexual minorities. LGBT health topics were mostly taught in lecture formats (Table 4).

**Table 4:** Disciplines and teaching format

**Discipline**	**N (lecturers)**	**Teaching format**
Human biology	2	Lectures and seminars with discussion on development of sex disorders, intersex, transitioning of transgender people
Primary health care directorate	1	Problem-based learning cases involve issues of gender and sexuality
Clinical laboratory sciences	1	One lecture on sexual orientation and HIV* & STIs+
Psychiatry	2	Lectures and seminars as “Introduction to Clinical Practice” and during Psychiatry module
Medicine	1	One lecture on HIV and men who have sex with men
		One elective clinical placement in a Men’s Health Clinic
Paediatrics	2	One lecture on paediatric endocrinology around disorders of sex development, else only “opportunistic teaching on ward rounds or if asked”
Family medicine	1	Elective special study module for 5th year students

*HIV: Human Immunodeficiency Virus, +STI: Sexually Transmitted Infection.

### Assessment

The assessment methods for LGBT health content varied across disciplines and lecturers. Often these assessment questions were incorporated into exams at the end of one teaching block, and not directly linked to the LGBT health teaching unit. Three respondents (33%) stated that they did not assess students at all. Among the remaining seven respondents, four used multiple choice questions and four open questions. Objective Structured Clinical Examinations (OSCE), useful tools to assess bedside manners and student-patient communication, were hardly used: only one respondent incorporated LGBT aspects into an OSCE. The two respondents who taught an elective module required their students to produce a report for assessment.

## Discussion

The results of this curriculum mapping show that there was no structured approach to teaching LGBT health related content in the MBChB curriculum at the University of Cape Town, and no LGBT health content in the curricula of the allied health sciences.

In 2012, only 10 academic educators taught some aspects of LGBT health (e.g. HIV in men who have sex with men) within the MBChB programme, but these were scattered across disciplines and clinical years. As a result, medical students repeatedly learned about some topics, albeit in a disconnected way, while other topics were not taught at all. Stigma, discrimination and social exclusion, crucial predictors of LGBT people’s health, were not addressed in any of the curricula.

The findings for the coverage of LGBT health related content in the MBChB curriculum are similar to results from other countries. In Obedin-Maliver’s review of US and Canadian medical schools [22] one third of schools did not teach any LGBT health related content in clinical years, while coverage in the remaining schools varied considerably. Similar to UCT’s MBChB curriculum, in 67% of schools LGBT health related content was mostly interspersed throughout the curriculum. Of note is that while issues around transitioning and sex reassignment surgery were the least taught in US and Canadian medical schools, UCT’s curriculum covered them more than other key LGBT health topics. This could be explained by the fact that Groote Schuur Hospital, UCT’s academic teaching hospital, has a dedicated transgender health service, which provides transgender advocacy and health education. Furthermore, teaching around sex and sexual development was well covered in pre-clinical Human Biology, as well as clinical Paediatrics. However, the extent to which these courses talked to the social realities and psychosocial needs of transgender people remained unclear.

The results highlight a number of gaps in the MBChB curriculum. First, none of the courses that addressed LGBT health related topics offered students the opportunity to engage with their own attitudes towards LGBT patients. Attitudes, knowledge and skills are interrelated, and all three need to be addressed to equip students with maximal competence in providing competent care for LGBT patients [25]. While there are no South African studies yet, research from other countries suggests that some medical students possess discriminatory and homophobic attitudes towards LGBT patients. For example, Jones et al. [26] reported that across 1132 students who were studying towards a health care degree, 30% felt uncomfortable treating a lesbian client, and 27% a gay male client. In the United Kingdom, 10 to 15% of medical students had negative or very negative attitudes towards male homosexual patients [27]. Recent South African research highlights that LGBT people often face discrimination by health care professionals when seeking health care. Lane et al. [17] report that all gay men in their study who visited clinics in the Soweto area experienced name-calling, ridiculing or other forms of discrimination. Similarly, 60% of transgender respondents in a study by Stevens [18] had negative experiences in public clinics. The non-governmental organisation OUT reports that 12% of gay men and lesbian women in Gauteng and 13% in KwaZulu-Natal delayed seeking treatment at clinics because of fear of discrimination, while 6% of participants in Gauteng and 5% in KwaZulu-Natal had been refused treatment because of their sexual orientation [28]. These findings are unsurprising given that 61% of South Africans think that society should not accept homosexuality^b^, and health professionals’ attitudes are clearly rooted in wider societal perceptions. Creating opportunities for medical students to engage with their own attitudes towards homosexuality can provide the space to challenge these societal assumptions. Kelley et al. [29] showed that students who received teaching on sexuality and LGBT health felt better equipped and more comfortable treating patients who identified as LGBT, and increasingly understood the clinical relevance of sexual orientation and gender identity. Such teaching could be incorporated into patient-physician communication and history-taking courses; additionally clinical educators should model non-judgmental care [30].

Second, the curriculum did not provide students with the knowledge or skills to address LGBT people’s specific health needs. For example, students were not taught how to adequately take sexual histories from, or provide safer sex information to LGBT patients. Given that in South Africa, the HIV prevalence among men who identify as gay and men who have sex with men is estimated between 34 and 49 per cent [8,31], this lack of knowledge is indefensible. History-taking and health prevention based on the heteronormative assumption that patients only have sex with people of the opposite sex risks not addressing the specific sexual risk behaviours of LGBT people, and misses the opportunity to educate LGBT patients about safer sex options. Taking inclusive sexual histories and acknowledging LGBT sexual behaviour is crucial to make LGBT patients feel comfortable and ensure that they receive appropriate preventative care [32]. In order to address the lack of teaching about key areas of concern for LGBT people’s health such as substance use, adolescent mental health, as well as health promotion and prevention with regards to non-communicable disease [33], Lock’s strategies to teach these topics within existing courses [30] can be a helpful starting point.

It is alarming that none of the allied health sciences curricula addressed LGBT health topics. There is not much evidence of allied health sciences LGBT health teaching in the international literature, but in an examination of dental school curricula, Anderson et al. [34] confirm the absence of LGBT health related teaching in 23 out of 30 US and Canadian dental schools, and thus highlight a crucial gap in the education of allied health professionals. Of particular concern is the absence of LGBT health related content in UCT’s postgraduate nursing curriculum, which aims to educate nurses in higher management positions. South Africa’s health system is based on primary health care and experiences a shortage of physicians [35]. With 113,000 nurses and only 16,000 physicians, a nurse-physician ratio of 7:1 ^[ibid]^, LGBT patients are more likely to interact with nurses than with physicians. For example, almost all HIV counselling and testing services are offered by nurses or counsellors [19]. The attitudes of nurses and allied health professionals towards LGBT patients are not well explored in literature, but research from Sweden [36]^: 386^] recommends that “more needs to be done to increase the positive attitudes among the nursing staff and students with neutral attitudes (neither positive nor negative attitudes) to enhance the wellbeing of homosexual persons”. Given that many South Africa nurses have highly conservative attitudes around contested topics such as sexual and reproductive health rights [37], it is likely that their attitudes towards LGBT patients, much like wider society’s^b^, are also often disapproving. The above-presented strategies to challenge these attitudes in students can be adapted and included in the curricula of nursing and other allied health professions.

This study is subject to some important limitations. First, not every teaching unit in the faculty responded to the survey. This might underestimate the amount of LGBT health topics currently taught. However, most units that did not respond have little thematic overlap with LGBT health related topics (e.g. clinical haematology). Furthermore, I assume that people who did not answer the survey were less likely to teach LGBT health related content and therefore saw less need to participate. Second, calculating a response rate for the survey was not possible, because the faculty does not keep a list of all academic educators teaching in the MBChB curriculum (some lecturers have permanent posts, while others are contracted to teach one specific course or seminar every semester). Third, I was unable to examine the lecture and seminar content beyond the listing of main topics due to limited resources. I recommend that future work includes audits of identified LGBT health lectures and a review of LGBT health related teaching material, in order to assess content and pedagogy in more detail.

## Conclusion

A survey of the curricula in UCT’s health sciences faculty showed that the MBChB curriculum did not address LGBT health related content in a structured, comprehensive way and that the curricula of the allied health sciences did not address LGBT health related content at all. Health professions students should understand how social exclusion, stigma and discrimination affect LGBT people’s health, and challenge their own attitudes and assumptions to become non-judgmental health care providers. LGBT health related topics need to be incorporated into the existing curricula in order to equip students to provide competent care to LGBT patients.

## Endnotes

^a^I acknowledge that sexual orientation and gender identity are fluid, and encompass more than the LGBT acronym. For example, a growing group of people have reappropriated the word ‘queer’ to identify their sexual orientation and/or gender identity. However, the majority of the current research evidence that I cite only focuses on LGBT identities, and I have therefore chosen to adhere to this terminology.

^b^According to a global study on societal acceptance of homosexuality, published by the Pew Research Center in June 2013. Available online at http://www.pewglobal.org/files/2013/06/Pew-Global-Attitudes-Homosexuality-Report-FINAL-JUNE-4-2013.pdf (accessed 19 Nov 2013).

## Competing interest

This project was financed by the author’s postdoctoral fellowship from the University Research Council at UCT. The author declares that she has no conflict of interest.

## Pre-publication history

The pre-publication history for this paper can be accessed here:

http://www.biomedcentral.com/1472-6920/13/174/prepub
